# Associations between Changes in Fat-Free Mass, Fecal Microbe Diversity, and Mood Disturbance in Young Adults after 10-Weeks of Resistance Training

**DOI:** 10.3390/microorganisms10122344

**Published:** 2022-11-26

**Authors:** Kristen S. Smith, Molly M. Morris, Casey D. Morrow, Josh R. Novak, Michael D. Roberts, Andrew Dandridge Frugé

**Affiliations:** 1 Department of Nutrition, Dietetics, and Hospitality Management, Auburn University, Auburn, AL 36849, USA; 2 College of Science and Mathematics, Auburn University, Auburn, AL 36849, USA; 3 Department of Cell, Developmental and Integrative Biology, University of Alabama at Birmingham, Birmingham, AL 35294, USA; 4 Department of Human Development and Family Sciences, Auburn University, Auburn, AL 36849, USA; 5 School of Kinesiology, Auburn University, Auburn, AL 36849, USA; 6 College of Nursing, Auburn University, Auburn, AL 36849, USA

**Keywords:** gut microbiome, resistance training, alpha diversity, profile of mood states

## Abstract

Background: The gut microbiome contributes to numerous physiological processes in humans, and diet and exercise are known to alter both microbial composition and mood. We sought to explore the effect of a 10-week resistance training (RT) regimen with or without peanut protein supplementation (PPS) in untrained young adults on fecal microbiota and mood disturbance (MD). Methods: Participants were randomized into PPS (n = 25) and control (CTL [no supplement]; n = 24) groups and engaged in supervised, full-body RT twice a week. Measures included body composition, fecal microbe relative abundance, alpha- and beta-diversity from 16 s rRNA gene sequencing with QIIME2 processing, dietary intake at baseline and following the 10-week intervention, and post-intervention MD via the profile of mood states (POMS) questionnaire. Independent samples *t*-tests were used to determine differences between PPS and CTL groups. Paired samples *t*-tests investigated differences within groups. Results: Our sample was mostly female (69.4%), white (87.8%), normal weight (body mass index 24.6 ± 4.2 kg/m^2^), and 21 ± 2.0 years old. Shannon index significantly increased from baseline in all participants (*p =* 0.040), with no between-group differences or pre-post beta-diversity dissimilarities. Changes in *Blautia* abundance were associated with the positive POMS subscales, Vigor and self-esteem-related-affect (SERA) (rho = −0.451, *p =* 0.04; rho = −0.487, *p =* 0.025, respectively). Whole tree phylogeny changes were negatively correlated with SERA and Vigor (rho = −0.475, *p =* 0.046; rho = −0.582, *p =* 0.011, respectively) as well as change in bodyfat percentage (rho = −0.608, *p =* 0.007). Mediation analysis results indicate changes in PD Whole Tree Phylogeny was not a significant mediator of the relationship between change in fat-free mass and total MD. Conclusions: Mood state subscales are associated with changes in microbial taxa and body composition. PD Whole Tree Phylogeny increased following the 10-week RT regimen; further research is warranted to explore how RT-induced changes in microbial diversity are related to changes in body composition and mood disturbance.

## 1. Introduction

The gastrointestinal (GI) tract is home to the largest collection of microbes in the human body [1] with a collective genome known as the gut microbiome. In recent years, research interest into the gut microbiome has vastly increased due to its considerable role in human health [2], including cytokine production [3], metabolism [4,5], inflammation [6], response to stress [7], and adaptations to exercise [8]. Greater microbial diversity is beneficial to host health and can be affected by lifestyle factors, including diet and exercise [9,10,11]. Certain commensal bacteria can produce neurotransmitters, such as serotonin [12,13]. Moreover, the gut microbiome affects signaling in the central nervous system via the gut-brain axis [14].

Consumption of whey or pea protein extract has been shown to increase commensal bacteria in the gut environment [15,16]. Protein supplements are often consumed by athletes [17] due to their efficacy in increasing strength and fat-free mass (FFM) body mass [18]. Cronin et al. reported altered diversity after 8-week supplementation with whey protein. Findings from this study also showed a trend towards increased microbial diversity following a combined aerobic and resistance training (RT) intervention in sedentary adults [19]. Similarly, in a human cross-sectional study, professional rugby athletes consuming higher protein diets displayed greater gut microbial diversity compared to lower FFM sedentary controls [20]. Alternatively, the gut microbiome may impact physical performance [11]. Estaki et al. reported correlations between microbial diversity and cardiorespiratory fitness [21]. Nay and colleagues demonstrated reduced muscle contractile function in mice fed antibiotics [22]. Several rodent studies have indicated that exercise alters gut microbial composition and function [4,23,24,25,26]. Exercise has been shown to alter microbial diversity in humans as well, although this research is limited [19,27].

Another suggested benefit of exercise is the improvement in emotional well-being and mood [28]. Mood disturbance is defined by feelings of distress or sadness, and symptoms of anxiety and depression [29]. Accordingly, mood-related symptoms are common in chronic diseases such as cancer, [30], diabetes [31], and HIV [32]. Participating in a 10-week RT program reduced depressive symptoms in adults with high risk for type 2 diabetes [33], and a 6-week combined aerobic and RT program improved mood disturbance in patients with HIV [34]. There are many proposed mechanisms, including increased serotonin production [35], improved activity of central nervous system [36], and increased neurogenesis [37].

Our previous investigation in an older adult population found that peanut protein (PP) supplementation did not alter gut microbiome diversity, however, it did increase genera with metabolically beneficial gene pathways [38]. Because of the similar physiological effects of the gut microbiome and exercise, the purpose of this study was to explore the effect of PPS during a 10-week RT regimen in untrained young adults on gut microbial diversity and mood disturbance. We also investigated if changes in alpha diversity explained the relationship between changes in FFM and mood disturbance via mediation analysis.

## 2. Materials and Methods

For this secondary analysis, we assessed diet, mood, and fecal microbiome composition in young female adults participating in a 10-week RT intervention. The university’s Institutional Review Board (IRB) approved the study protocol prior to recruitment (Protocol # 19-249 MR 1907) and this study was pre-registered as a clinical trial (NCT04707963; registered 13 January 2021). Detailed methods have been previously described [39].

### 2.1. Participants

Recruitment of participants occurred on campus and locally via emails, flyers, and word of mouth. Eligibility criteria included (1) 18–30 years old; (2) body mass index (BMI) <35 kg/m^2^; (3) not actively participating in RT more than one time per week in the preceding six months; (4) no known peanut allergy; (5) free of metal implants that could interfere with X-ray procedures; (6) no medically necessary radiation exposure for six months prior; (7) free of obvious cardiovascular or metabolic disease; (8) free of conditions contraindicating participation in exercise program or donation of muscle biopsy (i.e., blood thinners or blood clotting disorder); (9) could not be pregnant or trying to become pregnant. Eligible participants were informed of all study procedures prior to signing informed consent.

### 2.2. Study Design

For baseline (T1-1) procedures, participants reported to the Kinesiology Building at Auburn University for testing battery. First, a rapid pregnancy test was taken by female participants. Next, height and weight were measured using laboratory scales, with values rounded to the nearest 0.1 kg and 0.5 cm. Whole-body dual energy X-ray absorptiometry (DEXA) was utilized to determine body composition. Participants were randomized to either the peanut protein supplementation (PPS) or control (CTL) group. Each participant was provided with a three-day food log and stool specimen collection kit to complete and bring with them at the next appointment (T1-2). This next appointment occurred three days after T1-1 and included the first round of strength testing on bilateral leg press, barbell bench press, and hex-bar deadlift. Study staff trained and supervised participants on proper form throughout each training session. After completing the baseline strength assessment, the PPS group consumed their first supplement shake and CTL did not consume a nutritional shake but were provided water. The following ten weeks after T1-2 involved twice weekly training sessions for a total of 20 sessions. Each training session included (1) a warm-up of 25 jumping jacks + 10 bodyweight squats; (2) warm-up of 1 set of 10 repetitions (reps) @ 50% working weight, 1 set of 5 reps @ 75% working weight, and 1 set of 3 reps @ 90% working weight; and (3) 4 sets of 10 reps (high volume) or 5 sets of 6 reps (high load) at working weight. For the first 4 weeks, weekly loads were raised by ~5% for high volume day and ~9% for the high load day. Week 5 was reduced load training, consisting of 50% intensity for both high volume and high load training days. Loads were pre-programmed for all participants; however, rating of perceived exhaustion (RPE) was collected after each set to ensure appropriate loads were implemented throughout. During the last training session, strength testing was conducted on the same exercises previously discussed. Participants were provided another set of three-day food logs and stool collection kits for post-intervention testing (T-3), which was conducted 72 h after strength testing. The battery of tests conducted at baseline are repeated for T-3, including pregnancy test, height and weight, DEXA, and submission of food-logs and stool sample.

### 2.3. Peanut Protein Supplement

Participants in the PPS group were provided PBFit (BetterBody Foods, Lindon, UT, USA) to consume on non-workout days, and study staff would prepare the supplement and supervise the consumption on workout days. Participants were instructed to not consume the supplement as a meal replacement. The PPS supplement prepared by study staff included 16 ounces H_2_O and 75 g of the PBFit powder to provide 30 g/day protein, >9.2 g/day essential amino acids, and ~315 kcal. Amino acid content per daily serving has been described previously [39].

### 2.4. Dietary Analysis

Three-day food logs were completed by participants and included nutritional intakes for two weekdays and one weekend day prior to testing day. Normal dietary habits were encouraged throughout the duration of the study protocol, aside from the supplement for individuals in the PPS group. The Nutrition Data System for Research (NDSR; NDSR 2014; University of Minnesota) was used for food log entry and analyses [40]. Calories, micro- and macronutrients, and food group data from each time point represent the three-day average for the respective food log dates.

### 2.5. Fecal Sample Processing

Participants were given a commode specimen collection kit with a sterile collection tube and instructed to store sample in freezer until submission at next appointment. Upon receipt, samples were stored at −80 °C prior to batch processing. Fecal microbial DNA was isolated using Zymo Research kits (Irvine, CA, USA). DNA samples were prepared, and polymerase chain reaction (PCR) amplified the 250 base pair variable region 4 of 16 s rRNA. The PCR library was sequenced on the Illumina Miseq (San Diego, CA, USA) [41], with all further processing described previously [38]. The Quantitative Insight into Microbial Ecology (QIIME) suite, version 1.7 using DADA2 generated amplicon sequence variants (ASVs) [42,43]. Taxonomic assignments were made after UCLUST, with a 97% similarity threshold, clustered sequences, using the SILVA database version 132 [44]. ASVs with average abundance >0.005% were further processed and grouped by taxonomy. Each sample had at least 24,450 sequences per sample (Rarefaction curve; Supplemental Figure S1) and all Phred scores were >20 (Supplemental Figure S2).

### 2.6. Mood Disturbance

Mood disturbance (MD) was assessed using the abbreviated Profile of Mood States (POMS) rating scale. Individuals are asked to rate their mood using a 5-point Likert scale with responses including “not at all” to “extremely” based on 40 different mood states. This questionnaire measures seven mood dimensions: (1) tension/anxiety; (2) anger/hostility; (3) vigor/activity; (4) fatigue/inertia; (5) depression/dejection; (6) confusion/bewilderment; (7) self-esteem-related affect (SERA). The positive subscales are vigor and SERA, while the remaining five subscales are considered negative. To calculate total MD, the total negative subscales (tension + anger + fatigue + depression + confusion) are subtracted from the total positive subscales (vigor + esteem-related affect), with a constant (100) added to eliminate negative total MD scores. A higher score indicated greater MD.

### 2.7. Statistical Analysis

All statistical analysis was conducted using SPSS Version 25.0 (IBM Corp, Armonk, NY, USA) and statistical significance was set at *p <* 0.05. Data are presented as mean ± standard deviation throughout, unless otherwise indicated. Chi-square tests analyzed differences in categorical variables between groups. Spearman correlations were conducted in the whole group to explore relationships between changes in alpha diversity, changes in body composition, and POMS subscales. Independent samples *t*-test explored differences in continuous variables between intervention groups at baseline and follow-up. Paired samples *t*-tests assessed differences in diet, microbiome, body composition, and MD variables within intervention groups. Spearman correlations were performed within intervention groups to explore relationships between POMS subscales and the 25 most abundant genera detected in samples. Alpha diversity was measured using ASV count (Richness), PD Whole Tree Phylogeny, Simpson index, and Shannon index. Beta-diversity was measured using Bray–Curtis Dissimilarity and Unweighted and Weighted Unifrac distance metrics. Kruskal–Wallis one-way analysis of variance (ANOVA) tests with false discovery rate (FDR) corrections were performed to compare relative abundance of all ASVs at all phylogenic levels between intervention groups at both time points.

#### Mediation Analysis

To explore the mediating effect of change in alpha diversity on the relationship between FFM changes and MD, a simple mediation analysis was conducted in the whole sample using PROCESS [45]. The outcome variable was POMS total MD (*Y*), the predictor variable was change in FFM (*X*), and the mediator was alpha diversity, measured using PD Whole Tree Phylogeny (*M*). Sex and race were included as covariates due to confounding differences in these variables. Mediation analysis was conducted to explore indirect effects, with significant effects supported by the absence of zero within the confidence intervals.

## 3. Results

**Figure 1:**
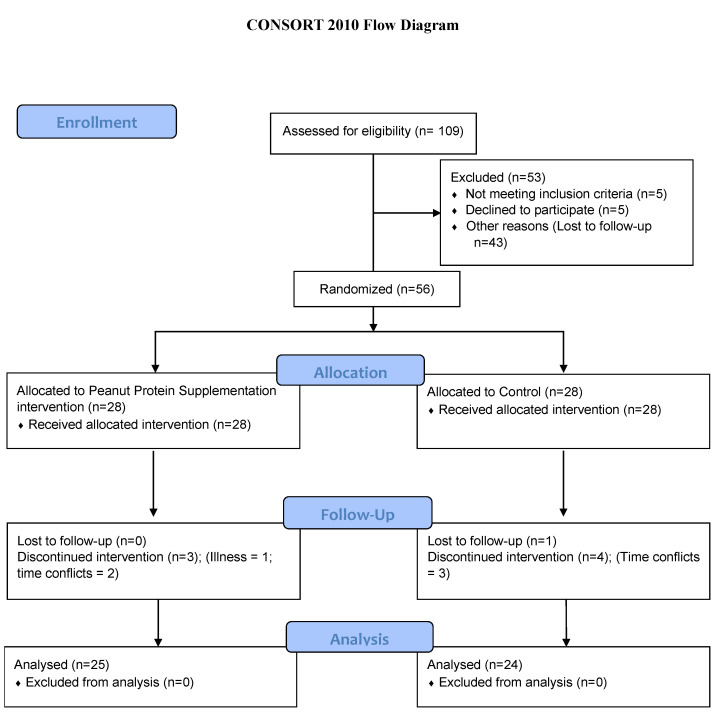
Consolidated Standards for Reporting Trials (CONSORT) Flow Diagram.

A total of 109 individuals expressed interest in participating and 56 individuals met eligibility criteria for enrollment in the study. Forty-nine participants completed the 10-week RT intervention. Figure 1 details participant retention and dropout. Table 1 reports demographic characteristics of the study sample and within the supplementation groups. Participants were mostly white (87.8%), female (69.4%), 21 ± 2.0 years of age and normal weight (BMI 24.6 ± 4.2 kg/m^2^). There were no significant differences in demographics between PPS and CTL groups.

Average daily intakes of macronutrients and dietary components are reported in Table 2. No differences in diet variables between groups were observed at baseline. From baseline to follow-up, participants significantly decreased fat consumption (from 73.7 ± 29.8 to 64.7 ± 24.6; *p =* 0.044). At follow-up the PPS group reportedly consumed more protein (91.1 ± 28.2 vs. 67.8 ± 24.7; *p =* 0.004) and fiber (23.8 ± 5.4 vs. 14.4 ± 7.7; *p <* 0.001) compared to the CTL group.

Table 3 presents body compositional changes with the intervention and differences between groups. Whole group changes included increased weight (74.1 ± 14.2 to 75.0 ± 18.1 kg; *p =* 0.024), increased FFM (47.2 ± 9.6 to 48.6 ± 10.1 kg; *p <* 0.001), decreased body fat (BF) % (31.3 ± 8.0 to 30.7 ± 7.8; *p =* 0.012), and increased BMI (24.6 ± 4.2 to 24.9 ± 4.4 kg/m^2^). Both groups significantly increased FFM (PP: 46.4 ± 9.5 to 47.7 ± 9.1, *p <* 0.001; CTL: 48.1 ± 9.8 to 49.4 ± 11.1; *p =* 0.002), while only the PPS group decreased BF% from baseline to follow-up (31.1 ± 8.0 to 30.2 ± 7.8, *p =* 0.014).

Beta diversity analyses indicate the 10-week RT intervention had no effect on microbiome composition (BC *p =* 1.000); however, differences were observed between males and females (BC *p =* 0.007 [Supplemental Figure S3]) and between PPS and CTL females (BC *p =* 0.009) with no group x time interaction (BC *p =* 0.792). Table 4 reports alpha diversity metric differences between intervention groups. Shannon Index significantly increased from baseline to follow-up in the whole group (4.97 ± 0.77 to 5.09 ± 0.71 *p =* 0.040). No differences were observed between intervention groups.

MD subscales are presented in Table 5. Participants in the PPS group reported greater feelings of Vigor compared to the CTL group (9.2 ± 4.2 vs. 6.6 ± 3.6; *p =* 0.023). Because of the between treatment group dissimilarity, relationships between mood disturbance subscales and bacterial genera relative abundance were explored by group (Supplemental Figure S4).

Within the PPS group, the genus *Subdoligranulum* positively associated with the SERA POMS subscale (rho = 0.458; *p =* 0.037) and *Romboutsia* associated with the Vigor subscale (rho = 0.465, *p =* 0.034). The Fatigue subscale was negatively correlated with *Ruminococcus* 1 and *Ruminococcus* 2 (rho = −0.465, *p =* 0.034; rho = −0.504, *p =* 0.02, respectively). *Anaerostipes* correlated with Anger subscale (rho = 0.540, *p =* 0.011) and total MD score (rho = 0.510, *p =* 0.018). Changes in richness were associated with changes in FFM (rho = 0.470, *p =* 0.049). PD Whole tree phylogeny changes were negatively correlated with change in %BF (rho = −0.608, *p =* 0.007), as well as SERA and Vigor (rho = −0.475, *p =* 0.046; rho = −0.582, *p =* 0.011) and positively associated with the Depression subscale (rho = 0.496, *p =* 0.036). Changes in *Blautia* abundance were associated with both positive POMS subscales, Vigor and SERA (rho = −0.451, *p =* 0.04; rho = −0.487, *p =* 0.025, respectively). Changes in *Subdoligranulum* abundance was positively correlated with Vigor subscale and changes in FFM (rho = 0.530, *p =* 0.014; rho = 0.469, *p =* 0.032, respectively).

Within the CTL group, the Anger subscale negatively correlated with *Roseburia* and *[Eubacterium] hallii group* (rho = −0.713, *p =* 0.004; rho = −0.540, *p =* 0.046, respectively), and positively correlated with *Bifidobacterium* and *Romboutsia* (rho = 0.540, *p =* 0.046; rho = 0.713, *p =* 0.004, respectively). The Depression subscale was negatively associated with *Roseburia* and *Lachnospiraceae_unclassified* (rho = −0.573, *p =* 0.032; rho = −0.539, *p =* 0.047, respectively). The Confusion subscale was positively correlated with *Coprococcus* 3 and *Anaerostipes* (rho = 0.537, *p* = 0.047; rho = 0.578, *p* = 0.031, respectively). The Fatigue subscale was positively associated with changes in both PD Whole Tree Phylogeny and Simpson (rho = 0.594, *p =* 0.019; rho = 0.678, *p =* 0.006). Changes in *Subdoligranulum* abundance was negatively correlated with the Anger, Fatigue, and Depression subscales (rho = −0.540, *p =* 0.046; rho = −0.734, *p =* 0.003; rho = −0.551, *p =* 0.041, respectively). In the CTL group, no correlations were observed between changes in top 25 genera and body composition.

**Figure 2:**
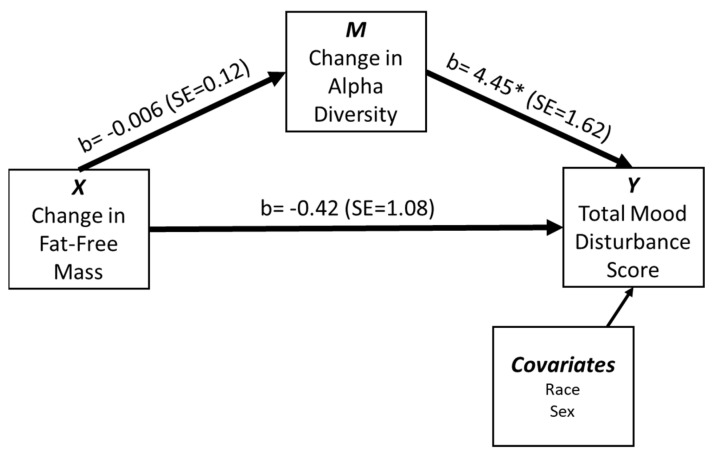
Simple mediation of Fat-Free Mass changes (X) on Total Mood Disturbance Score (Profile of Mood States) with Change in alpha diversity (as measured by PD Whole Tree Phylogeny) as a mediator. Race and sex are covariates in this model. Unstandardized regression coefficients are reported with standard error values; * *p-*value < 0.05.

Because of the relationships between POMS subscales and body composition changes, and POMS subscales and alpha diversity, we explored the mediating effects of the gut microbiome on the relationship between body composition changes and MD. The outcome variable was POMS total MD (*Y*), the predictor variable was change in FFM (*X*), and the mediator was PD Whole Tree Phylogeny (i.e., alpha diversity) (*M*). Results from the simple mediation analysis (Figure 2) indicate changes in alpha diversity did not mediate the relationship between changes in FFM and total MD (b = −0.449, t (29) = −0.374, *p =* 0.711). While change in FFM is not significantly associated with PD whole tree phylogeny (b = −0.006, t (29) = −0.049, *p =* 0.9610), the additional covariate of race improved the model (b = −0.958, t (29) = −0.242, *p =* 0.004). Furthermore, change in PD Whole Tree Phylogeny is significantly associated with total MD (b = 4.45, t(28) = 2.754, *p =* 0.0102), although neither the sex or race covariate significantly improved the model (*p <* 0.05 for both). The indirect effect of FFM on total mood disturbance was not significant (Indirect effects = −0.0273, SE = 0.708, 95% CI [−1.45, 1.54]). These results suggest FFM was not associated with change in alpha diversity (PD whole tree phylogeny) or total MD, but a direct association did exist between alpha diversity changes and total MD.

## 4. Discussion

This study explored the effects of PPS on diet, gut microbiota, and mood disturbance after 10 weeks of RT. We observed an increase in alpha diversity as measured by Shannon Index in the whole group and no differences between intervention groups. Additionally, several genera were related to POMS subscales. These results indicate RT can improve microbial diversity in younger untrained adults, however, only one measure of alpha diversity was improved. After a 6-week endurance-based training regimen in obese and lean participants, Allen et al. found beta-diversity changes were dependent on BMI and any changes in the microbiota were reversed after terminating training [27]. Similarly, Cronin et al. reported that moderate-intensity mixed aerobic and RT for 8 weeks resulted in modest increases in microbial diversity although this only trended towards significance [19]. However, the training protocols in these studies differed from ours in the incorporation of aerobic exercise, which could explain differences in our results. Notably, aerobic exercise can modify microbial diversity and abundance of certain genera, although investigations into the effects of resistance or strength training are limited. Bycura et al. compared the effects of aerobic training and RT throughout 8-week regimens, and observed no change in the microbiome from RT, while aerobic training had transient effects on diversity [46].

In our study, *Roseburia* was negatively associated with the Anger and Depression subscales in the CTL group. In anorexia nervosa patients, decreased fecal *Roseburia* has been reported compared to healthy controls [47]. *Roseburia* is known to produce butyrate, a short chain fatty acid (SCFA) that sustains colonocytes and is beneficial for inflammation and intestinal barrier function [48]. Butyrate has also shown anti-depressive properties in mice by reducing behaviors related to cognitive and social impairments and low energy [49,50]. In the context of PA, individuals with greater aerobic fitness produce more butyrate [21]. While SCFAs were not measured in the current study, two negative subscales (Anger and Depression) had an inverse relationship with the genus *Roseburia* which could be due to the inferred production of SCFAs. Alternatively, obese children showed increased levels of *Roseburia* after participating in a 12-week endurance and RT program, in similar abundance to the healthy control group [51]. These results suggest physical activity can improve the composition of the gut microbial community, as determined from relative abundance of commensal bacteria, which may lead to mood-related improvements via the GBA.

*Anaerostipes* is another genus known to produce butyrate [52]; however, we observed a positive relationship between this genus and Anger and total MD in the PPS group, while the CTL group showed a positive relationship between *Anaerostipes* and Confusion. These results differ from the butyrate-producing *Roseburia*, and other previous evidence indicating *Anaerostipes* improves gut barrier integrity and has anti-inflammatory properties [53]. Verheggen et al. found increased *Anaerostipes* following 8 weeks of aerobic training in obese individuals, although no changes were seen in alpha diversity metrics, similar to the results found herein [54].

The genus *Romboutsia* was positively associated with the Vigor subscale in the PPS group, but positively associated with Anger and Fatigue subscale in the CTL group. Species within *Romboutsia* genus are able to synthesize some B vitamins, metabolize carbohydrates, and ferment amino acids [55], supporting its potentially beneficial role in human health. Alternatively, *Romboutsia* is suggested to inhibit enzymatic steps along the serotonin production pathway [56], which plays an important role in mood [57]. On the other hand, a single exercise bout in endurance athletes showed an increase in abundance of *Romboutsia* [58], while decreased *Romboutsia* was observed following exercise and vitamin C supplementation in a rat model [59]. These differences may be due to lack of precision; thus, species-level genomic analysis may reveal more insight into the impact of the *Romboutsia* genus on human health.

Consumer trends indicate plant-based protein is increasing in popularity [60], but are unproven in comparison to the benefits of whey protein [61,62,63,64]. PPS is equivalent to meat and eggs for promoting health and growth in humans and equally as digestible [65,66], and PPS has the additional benefits of fiber and phytonutrients [67]. Because study protocol advised the CTL group from changing diet, the participants in the PPS group consumed more fiber and protein. Dietary fiber can improve microbial composition by supplying energy to commensal bacteria [68,69], while the effects of protein on microbiota display mixed results [70,71]. Interestingly, the participants in the PPS group experienced more feelings of vigor compared to CTL. A study showed mood improvements after including a fiber-containing cereal bar at breakfast compared to no breakfast meal [72]. The higher fiber content in the PPS shake could help explain the higher vigor levels within our sample.

### Limitations

While this study provides initial insight into PPS during RT and the health benefits associated, it is not without limitations. First, there is lack of diversity with participants given that 69.4% were female and 87.8% were white. More females than males were purposefully recruited for this training study due to the substantial lack of investigation into female populations in the sports and exercise field [73]. Subsequent exploration should include a more diverse sample to extrapolate findings to the general population. Secondly, our study design prevented us from exploring the differences between whey (or another animal-based protein) and peanut protein. Future research should compare the effects of PPS to other well-studied protein sources to inform recommendations on plant-based protein supplements. Moreover, collection of fecal samples occurred at various times during the day and circadian shifts in the microbiome could account for minimal differences observed [74]. Additionally, participants were responsible for keeping food logs, and human error could occur with self-reported data. Lastly, this secondary analysis of a relatively small sample is not adequately powered to establish conclusions from our current results. Further investigation is warranted to explore the effects of PPS on mood disturbance and gut microbiota in adequately powered samples.

## 5. Conclusions

Physical activity has long been suggested as beneficial to mood and affect, in part due to increased production of serotonin and other neurotransmitters [75]. Specifically, RT has been shown to reduce anxiety or depression symptoms, improve self-esteem-related affect, and alleviate fatigue [76]. The gut microbiome is a novel target that can improve mood and mental health [77]. Because of this interconnected relationship, improving composition of the microbiome and increasing physical activity are mechanisms by which mood can be improved with compounding health benefits [78,79]. Herein, we observed changes in microbial taxa related to body compositional changes and mood state subscales. We also found increased microbial diversity following a 10-week RT program, and microbial diversity may have mediated the relationship between mood disturbance and body composition changes. These results support previous research indicating changes in gut microbial composition following exercise, and the relationship between the gut microbiome and mood disturbance. Given these observations, opportunities to encourage and maintain behavior change (e.g., resistance and/or endurance training exercise) through increased dietary protein and fiber; the addition of probiotic strains capable of improving mood and reducing anxiety could add synergistic effects as well. Further research is necessary to explore gut microbial responses to resistance training and their compounding effects on mood.

## Figures and Tables

**Table 1:** Demographic characteristics of young adults participating in a 10-week resistance training study by supplementation intervention groups.

	All (n = 49)	Peanut (n = 25)	Control (n = 24)	*p-*Value
Age *	21.1 (2.0)	21.4 (2.2)	20.8 (1.7)	0.358
Baseline Body Mass Index *	24.6 (4.2)	23.8 (3.7)	25.4 (4.7)	0.197
Sex ^#^				0.686
Female	34 (69.4)	18 (72)	16 (66.7)	
Male	15 (30.6)	7 (28)	8 (33.3)	
Hispanic ^#^				0.368
Cuban	1 (2)	1 (4)	0 (0)	
Another Hispanic, Latino, or Spanish Origin	1 (2)	1 (4)	0 (0)	
No Hispanic, Latino, or Spanish Origin	47 (95.9)	23 (92)	24 (100)	
Race ^#^				0.572
Asian	1 (2)	1 (4)	0 (0)	
Black or African American	4 (8.2)	2 (8)	2 (8.3)	
White	43 (87.8)	21 (84)	22 (91.7)	
More than one race	1 (2)	1 (4)	0 (0)	
Education ^#^				0.210
HS Grad	3 (6.1)	0 (0)	3 (12.5)	
Some College	35 (71.4)	19 (76)	16 (66.7)	
AA or AS	1 (2)	5 (20)	1 (4.2)	
Bachelors	7 (14.3)	1 (4)	2 (8.3)	
Masters	3 (6.1)	0 (0)	2 (8.3)	
Baseline Body Mass Index Category ^#^				0.536
Underweight	2 (4.1)	2 (8)	0 (0)	
Normal	32 (65.3)	16 (64)	16 (66.7)	
Overweight	10 (20.4)	5 (20)	5 (20.8)	
Obese	5 (10.2)	2 (8)	3 (12.5)	
Marital Status ^#^				0.966
Single	45 (91.8)	23 (92)	22 (91.7)	
Married or Domestic Partnership	4 (8.2)	2 (8)	2 (8.3)	

* Presented as mean (SD); ^#^ presented as N (%).

**Table 2:** Dietary components between supplementation intervention groups of young adults participating in a 10-week resistance training study.

	*p-*Value *	All (n = 49)	*p-*Value ^#^	Peanut (n = 25)	*p-*Value ^#^	Control (n = 24)	*p-*Value ^#^
Calories (kcal)			0.285		0.640		0.255
PRE	0.187	1640.0 (611.7)		1753.6 (665.9)		1521.7 (538.1)	
POST	0.063	1547.5 (545.7)		1689.0 (655.1)		1400.2 (359.4)	
Protein (g)			0.385		0.120		0.825
PRE	0.318	74.8 (34.4)		79.7 (35.5)		69.8 (33.2)	
POST	**0.004**	79.7 (28.8)		91.1 (28.2)		67.8 (24.7)	
Carbohydrate (g)			0.545		0.956		0.327
PRE	0.454	170.0 (84.4)		178.9 (88.9)		160.7 (80.2)	
POST	0.102	163.7 (62.5)		178.0 (74.6)		148.7 (43.3)	
Fat (g)			**0.044**		0.076		0.300
PRE	0.134	73.7 (29.8)		79.9 (28.3)		67.1 (30.6)	
POST	0.230	64.7 (24.6)		68.8 (29.7)		60.3 (17.5)	
Fiber (g)			**0.000**		**0.000**		0.309
PRE	0.257	13.5 (6.3)		14.5 (6.5)		12.5 (6.1)	
POST	**0.000**	19.1 (8.1)		23.8 (5.4)		14.4 (7.7)	
Sugar (g)			0.215		0.803		0.129
PRE	0.810	58.0 (44.5)		56.5 (39.7)		59.6 (49.8)	
POST	0.309	51.5 (31.5)		56.1 (38.0)		46.8 (23.1)	

* Between-group *p-*value; ^#^ within-group *p-*value; bold font indicates statistical significance.

**Table 3:** Body composition metrics and differences between supplementation intervention groups of young adults participating in a 10-week resistance training study.

	*p*-Value *	All (n = 49)	*p*-Value ^#^	Peanut (n = 25)	*p*-Value ^#^	Control (n = 24)	*p*-Value ^#^
Weight			**0.024**		0.122		0.108
PRE	0.370	74.1 (17.2)		71.9 (14.2)		76.4 (19.9)	
POST	0.374	75.0 (18.1)		72.7 (13.3)		77.4 (22.1)	
Fat Mass			0.615		0.236		0.742
PRE	0.451	23.8 (10.2)		22.7 (8.5)		24.9 (11.8)	
POST	0.359	23.7 (10.5)		22.3 (7.8)		25.1 (12.7)	
Fat Free Mass			**0.000**		**0.000**		**0.002**
PRE	0.549	47.2 (9.6)		46.4 (9.5)		48.1 (9.8)	
POST	0.549	48.6 (10.1)		47.7 (9.1)		49.4 (11.1)	
Body Fat Percentage			**0.012**		**0.014**		0.264
PRE	0.823	31.3 (8.0)		31.1 (8.0)		31.6 (8.2)	
POST	0.687	30.7 (7.8)		30.2 (7.8)		31.2 (8.0)	
Body Mass Index			**0.029**		0.103		0.155
PRE	0.197	24.6 (4.2)		23.8 (3.7)		25.4 (4.7)	
POST	0.219	24.9 (4.4)		24.1 (3.3)		25.7 (5.2)	

* Between-group *p*-value; ^#^ within-group *p*-value; bold font indicates statistical significance.

**Table 4:** Alpha diversity measures between intervention groups of young adults in a 10-week resistance training study.

	*p*-Value *	All (n = 49)	*p-*Value ^#^	Peanut (n = 25)	*p-*Value ^#^	Control (n = 24)	*p-*Value ^#^
Richness			0.688		0.355		0.439
PRE	0.108	122.0 (34.2)		111.0 (32.0)		131.9 (40.7)	
POST	0.302	120.5 (37.2)		116.6 (37.4)		128.6 (30.0)	
Whole Tree Phylogeny			0.594		0.224		0.233
PRE	0.057	9.95 (2.55)		9.12 (2.29)		10.96 (2.55)	
POST	0.294	10.08 (2.50)		9.60 (2.75)		10.67 (2.09)	
Shannon Index			**0.040**		0.062		0.377
PRE	0.263	4.97 (0.77)		4.85 (0.87)		5.13 (0.61)	
POST	0.432	5.09 (0.71)		5.01 (0.83)		5.20 (0.54)	
Simpson Index			0.165		0.262		0.438
PRE	0.376	0.93 (0.06)		0.92 (0.08)		0.94 (0.03)	
POST	0.439	0.93 (0.07)		0.93 (0.09)		0.94 (0.03)	

* Between-group *p-*value; ^#^ within-group *p-*value; bold font indicates statistical significance.

**Table 5:** Mood disturbance after a 10-week resistance training study between supplementation intervention groups.

	All (n = 49)	Peanut (n = 25)	Control (n = 24)	Between Group *p-*Value
Tension	2.8 (2.9)	3.2 (3.4)	2.5 (2.2)	0.400
Anger	0.4 (1.0)	0.4 (1.3)	0.3 (0.6)	0.712
Fatigue	3.1 (3.2)	2.9 (3.8)	3.3 (2.5)	0.690
Depression	0.5 (1.2)	0.5 (1.5)	0.5 (0.8)	0.955
Esteem-Related Affect	16.6 (3.3)	17.4 (3.3)	15.8 (3.1)	0.103
Vigor	7.9 (4.1)	9.2 (4.2)	6.6 (3.6)	**0.023**
Confusion	1.5 (2.1)	1.7 (2.0)	1.3 (2.1)	0.562
Total Mood Disturbance	83.8 (10.9)	82.2 (12.2)	85.5 (9.1)	0.286

Bold font indicates statistical significance.

## Data Availability

All data can be obtained from the corresponding author (kss0034@auburn.edu).

## Supplementary Materials

The following supporting information can be downloaded at: https://www.mdpi.com/article/10.3390/microorganisms10122344/s1, Figure S1: Rarefaction curve of alpha diversity (PD whole tree phylogeny) by sample;; Figure S2: Phred scores of study samples (n = 98) indicating >99.9% accuracy; Figure S3: Dissimilarity plots comparing males (blue squares) and females (red circles). (a) Bray Curtis, p = 0.007; (b) Unweighted Unifrac, p = 0.005; Figure S4: Spearman correlations between POMS subscales and top 25 genera by intervention group. Highlighted cells indicate *p* < 0.05.
