# Supplementary material for: Associations between Changes in Fat-Free Mass, Fecal Microbe Diversity, and Mood Disturbance in Young Adults after 10-Weeks of Resistance Training
Source: Microorganisms. 2022 Nov 26;10(12):2344. doi: 10.3390/microorganisms10122344 (PMC9785032; doi:10.3390/microorganisms10122344)
Supplement: Supplementary file 1 [file microorganisms-10-02344-s001.zip › microorganisms-2010882-supplementary.pptx]

## Slide 1
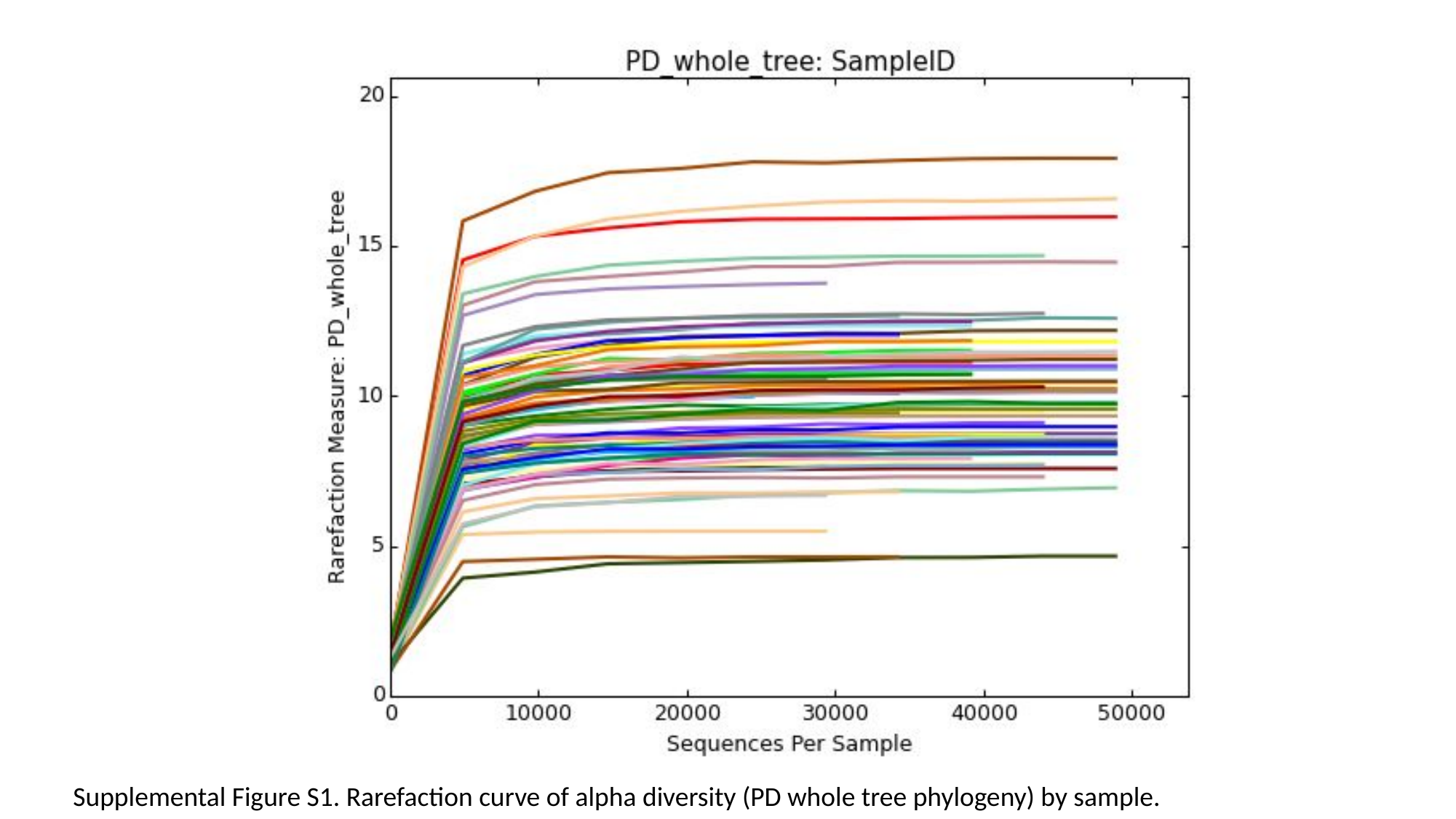

Supplemental Figure S1. Rarefaction curve of alpha diversity (PD whole tree phylogeny) by sample.

## Slide 2
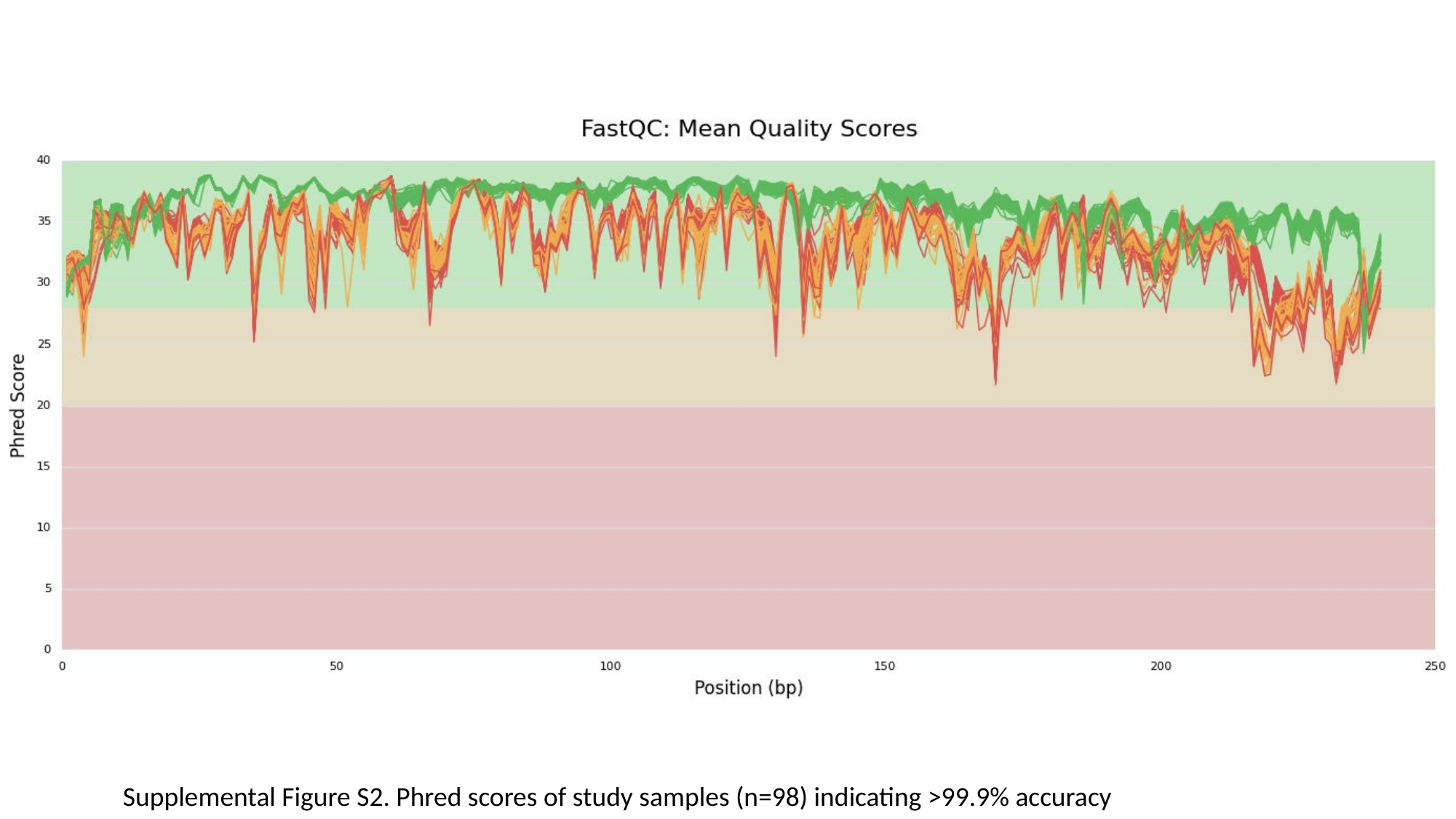

Supplemental Figure S2. Phred scores of study samples (n=98) indicating >99.9% accuracy

## Slide 3
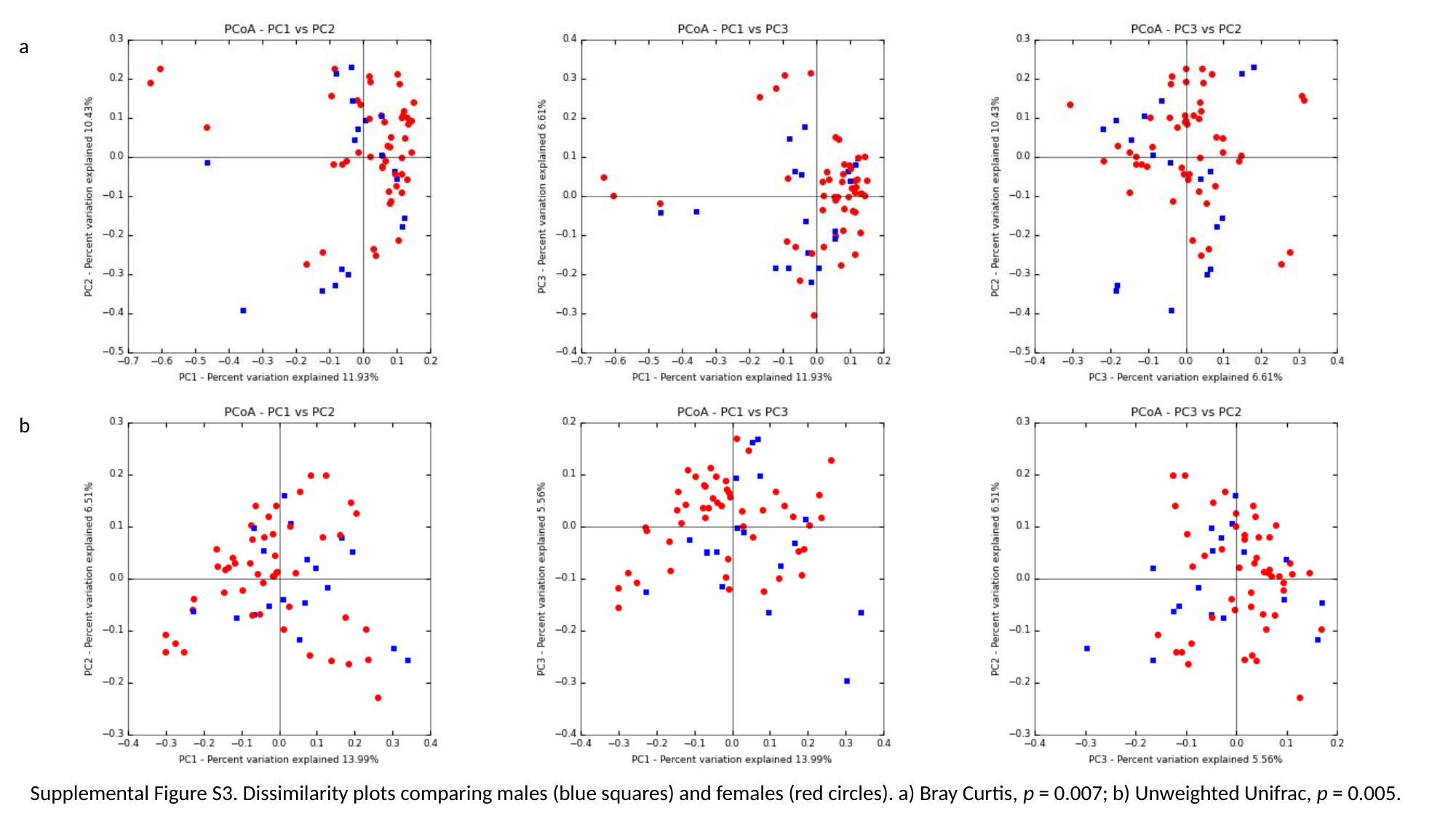

a
b
Supplemental Figure S3. Dissimilarity plots comparing males (blue squares) and females (red circles). a) Bray Curtis, p = 0.007; b) Unweighted Unifrac, p = 0.005.

## Slide 4
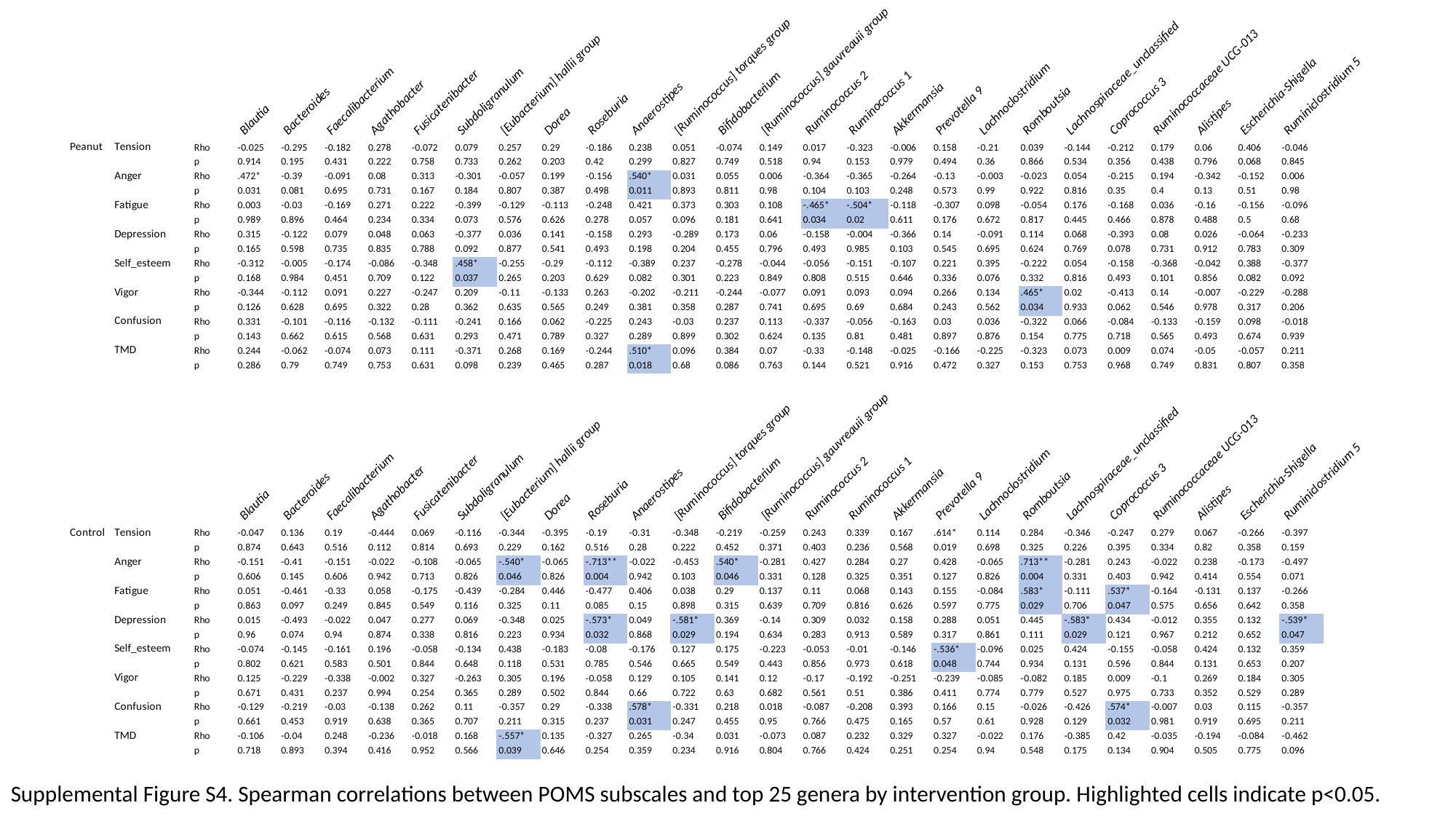

Supplemental Figure S4. Spearman correlations between POMS subscales and top 25 genera by intervention group. Highlighted cells indicate p<0.05.
